# Retrieval of Vegetation Indices Related to Leaf Water Content from a Single Index: A Case Study of *Eucalyptus globulus* (Labill.) and *Pinus radiata* (D. Don.)

**DOI:** 10.3390/plants10040697

**Published:** 2021-04-05

**Authors:** Juan Villacrés, Andrés Fuentes, Pedro Reszka, Fernando Auat Cheein

**Affiliations:** 1 Department of Electronic Engineering, Universidad Técnica Federico Santa María, Av. España 1680, Valparaiso 2390123, Chile; juan.villacres@sansano.usm.cl; 2 Department of Industrial Engineering, Universidad Técnica Federico Santa María, Av. España 1680, Valparaiso 2390123, Chile; andres.fuentes@usm.cl; 3 Faculty of Engineering and Sciences, Universidad Adolfo Ibáñez, Santiago 7941169, Chile; pedro.reszka@uai.cl

**Keywords:** fuel moisture content, vegetation indices, spectral reflectance, *Eucalyptus globulus*, *Pinus radiata*

## Abstract

The vegetation indices derived from spectral reflectance have served as an indicator of vegetation’s biophysical and biochemical parameters. Some of these indices are capable of characterizing more than one parameter at a time. This study examines the feasibility of retrieving several spectral vegetation indices from a single index under the assumption that all these indices are correlated with water content. The models used are based on a linear regression adjusted with least squares. The spectral signatures of *Eucalyptus globulus* and *Pinus radiata*, which constitute 97.5% of the forest plantation in Valparaiso region in Chile, have been used to test and validate the proposed approach. The linear models were fitted with an independent data set from which their performance was assessed. The results suggest that from the Leaf Water Index, other spectral indices can be recovered with a root mean square error up to 0.02, a bias of 1.12%, and a coefficient of determination of 0.77. The latter encourages using a sensor with discrete wavelengths instead of a continuum spectrum to estimate the forestry’s essential parameters.

## 1. Introduction

The use of sensors operating in the electromagnetic (EM) spectrum has allowed the estimation of several biophysical parameters in forest and urban environments [1,2]. Such estimates employ narrow bands of the EM spectrum, spectral transformations (e.g., derivatives, continuum removal, wavelets), or vegetation indices (VIs). The latter has been used in qualitative and quantitative studies of vegetation on a temporal and spatial scale [3,4]. Vegetation indices are structured from a limited set of wavelengths; VIs attempt to maximize sensitivity to a biophysical parameter while minimizing adverse effects (e.g., atmospheric composition and variations in canopy background) [5].

Some of the spectral indices are used to predict more than one parameter at a time, on scales ranging from leaf level to canopy level, and even on global scales. For example, one of the most common VI, the Normalized Difference Vegetation Index (NDVI), is used in applications to assess biomass, phenology, Leaf Area Index, plant growth, among others [2,5]. Another spectral index is the Double Difference Index (DDI), developed to evaluate chlorophyll levels, but it has also presented a high correlation with the equivalent water thickness [6,7]. Li and coworkers evaluated several chlorophyll-sensitive spectral indices to recover the nitrogen content in the vegetation [8]. Since each individual of a given plant species will have a specific spectral signature which depends on its biophysical and biochemical state and, therefore, on environmental conditions, it can be argued that, through the use of spectral reconstruction techniques based on data analysis, the characterization of several vegetation parameters can be performed from a single vegetation index.

In our previous work [9], we studied the correlation of several vegetation indices with the water content in the leaves of *Eucalyptus globulus* and *Pinus radiata*. Given the coefficient of determination values, we hypothesized that it is possible to estimate some spectral indices using only the most suitable one used to recover the moisture content. In this brief, 18 spectral indices related to water content are studied, and the most suitable one—from a water content perspective—is selected to recover the remaining ones. To validate this hypothesis, we carried out a dehydration process and worked with two data sets, one for fitting and the other for validating the model. The specific objectives of this study are (1) to develop a model to select the most appropriate index to recover moisture content; (2) to estimate the remaining seventeen water-related spectral indices from the index chosen in (1); and (3) to evaluate the performance of the model in terms of mean square error, coefficient of determination, and percent bias.

## 2. Materials and Methods

**Figure 1:**
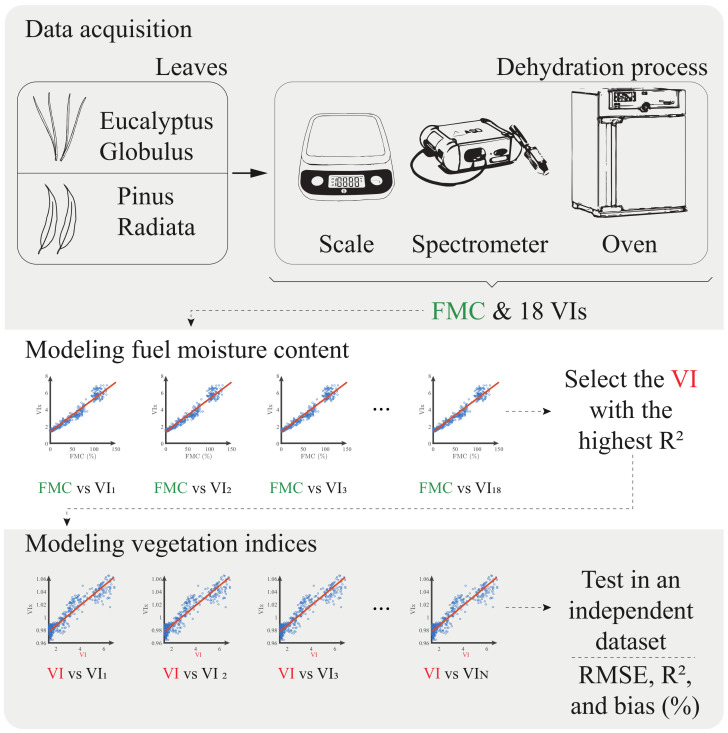
General scheme of the measurement and modeling process of the vegetation indices.

Figure 1 represents the work-flow followed in this study. It began with the acquisition stage, where leaves were collected and their reflectance spectrum measured. With this information, the fuel moisture content (FMC) and the eighteen vegetation indices were obtained. Then, the relationship of the fuel moisture content and the vegetation indices was expressed in terms of the coefficient of determination ($R^{2}$). The index with the highest $R^{2}$ was selected as suitable, and then the models were obtained to recover the rest of the indices from the selected one. Finally, a validation stage was carried out with an independent dataset. Each of these stages is detailed below.

### 2.1. Leaf Sampling

In the region of Valparaiso, located in central Chile, a field sampling of *Pinus radiata* and *Eucalyptus globulus* leaves was carried out. These leaves were collected from the lower part of the canopy during four measurement campaigns. The months in which these samples were obtained were July, August and September 2018, and the last one in January 2020. In the first three campaigns, a total of 90 samples were obtained, while in the last one, 27 samples were collected (per each species). This separation is due to the fact that this last data set will be used to evaluate the performance of the models.

Once the branches were cut from the trees, they were stored in plastic bags to minimize aging effects. The samples were taken to the laboratory in less than one hour to start the measuring process.

### 2.2. Reflectance Measurement and Dehydration Process

The *Eucalyptus* leaves were detached individually from the branches; on the contrary, as *Pinus* needles have an acicular shape, they were arranged in batches of approximately 5 g. At this point, the leaves were considered alive, their masses were registered with a balance and their reflectance was recorded with the ASD spectrometer. To perform the spectral measurement, each leaf was placed on a panel with constant reflectance throughout the spectrum. Then the contact probe, illuminated with a tungsten filament, was placed over the samples, and the spectral signature of each sample was recorded three times. The spectrometer was calibrated with a Spectralon before use, and after 30 measurements.

Since the models to be used depended on the variation of water content in the leaves, a dehydration process was carried out to have a wide range of samples with different moisture content levels. This process consisted of placing the leaves in a drying oven at 65 ${}^{{}^{\circ}}$C for 15 min for *Eucalyptus* leaves and 60 min for *Pinus* needles. After the drying process, the spectrum and mass were measured. This dehydration was repeated a total of three times, following the guidelines presented in [9,10]. Finally, to obtain the completely dry leaf mass, a 24-h drying process was carried out. In summary, five mass and reflectance values were measured for each vegetation sample. Table 1 lists the technical characteristics of the equipment used in this procedure. A general scheme of the measurement process is given at the top of Figure 1.

### 2.3. Moisture Content and Vegetation Indices

Once the measurement process was complete, the FMC was calculated as follows:

(1) $$FMC=\frac{W_{f,t}-W_{d}}{W_{d}}$$

where $W_{f,t}$ is the weight of the leaf at the time *t*, and $W_{d}$ is the dried leaf’s mass. Several researches have developed spectral indices with a high correlation with the moisture content. In this regard, we selected 18 vegetation indices previously reviewed in [9]. The complete list of indices is shown in Table 2.

### 2.4. Vegetation Index Retrieval

As stated in Section 1, this study aimed to retrieve water-related vegetation indices from a single index. Therefore, it was crucial to determine which index will be the basis for recovering the remaining 17. In this regard, the FMC was correlated from each spectral index using a linear model as shown in Equation (2):

(2) $$FMC=\alpha_{1,x}VI_{x}+\alpha_{2,x}$$

where $\alpha_{1,x}$, $\alpha_{2,x}$ are the parameters of the linear model fitted with least squares, the suffix $x\in \left\{1,2,3,\ldots ,18\right\}$ represents each one of the indices in Table 2. The model with the highest coefficient of determination is selected and named with suffix *H*. The FMC of the selected index $VI_{H}$ is equalized to each of the remaining spectral index models (indicated as $VI_{N}$) from this model stack. The final linear model with input $VI_{H}$, and the output each of the resting 17 indices is shown in Equation (3):

(3) $$VI_{N}=\frac{1}{\alpha_{1,N}}\left(\alpha_{1,H}\cdot VI_{H}+\alpha_{2,H}-\alpha_{2,N}\right)$$

### 2.5. Model Evaluation Metrics

The validation stage was conducted on an independent data set. The performance of the linear models was tested based on three metrics: coefficient of determination $R^{2}$, Root Mean Square Error (RMSE) as shown in Equation (4); and percentage bias (bias), see Equation (5). The first indicated how well the actual and predicted values of the VI fitted together. The second quantified the error made in the prediction; the lower the RMSE value, the better the model. The percentage bias indicated the estimated data’s tendency to be larger or smaller than their actual values. Positive bias values represented overestimation, and negative bias values represented underestimation [25].

(4) $$RMSE=\sqrt{\frac{1}{n}\sum_{i=1}^{n}{\left({\hat{VI}}_{i}-{VI}_{i}\right)}^{2}}$$

(5) $$bias=\frac{VI-\hat{VI}}{VI}\times 100\%$$

where ${\hat{VI}}_{i}$ is the prediction of the *i*th—vegetation index sample, $VI_{i}$ is the actual value of the *i*th—vegetation index, and *n* is the number of samples.

### 2.6. Non-Water Content Related VIs

We also estimated five vegetation indices—different from the 18 VIs chosen in Table 2—based on the index selected in Section 2.4. These VIs provided information on nitrogen content, anthocyanin, and carotenoid content. Besides, we included the most widespread VI in vegetation assessment, the NDVI [26]. Table 3 shows the acronym, formula, and reference information for each index.

## 3. Results

**Figure 2:**
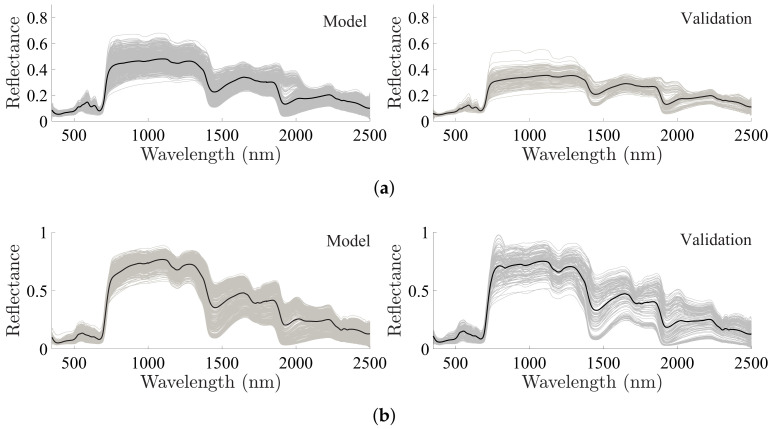
Set of samples used for fitting and validating the models. In black line the average reflectance spectra and in grey the reflectance of the individual samples. Where: (**a**) *Pinus radiata* and (**b**) *Eucalyptus globulus*.

As reported in Section 2, the data from the first three measurement campaigns were used to fit the models, while the last one was used to validate them. The time between the measurement of these two sets was 15 months and thus they were considered independent sets. The first column of Figure 2 presents the group used in the linear modeling, while the second column represents the validation set. The dehydration process resulted in a variation of the FMC between 0% when the leaves were dry (after 24 h in the oven), and a maximum of 157.68% and 180.04% for *Pinus* needles and *Eucalyptus* leaves, respectively. Nevertheless, we limited our work to water contents higher than 30% and 50% for *Pinus* and *Eucalyptus*, respectively. Those thresholds were defined according to the results obtained in [31,32], for *Pinus* and *Eucalyptus* species. These values were obtained under drought stress conditions; lower values of water content caused the plant to show traits of mortality [31].

### 3.1. Model Fitting

Table 4 shows that the maximum value of $R^{2}$ for both species was 0.8699, and was obtained with the Leaf Water Index (LWI) for *Eucalyptus globulus*. Also, this same index for *Pinus radiata* was the highest with $R^{2}$ = 0.7742. Thus, the LWI was selected as a suitable index to retrieve the rest of the indices. As supplementary material, the reader can refer to Appendix A for the variation of the 18 vegetation indices as function of FMC for *Pinus radiata* and *Eucalyptus globulus*.

**Figure 3:**
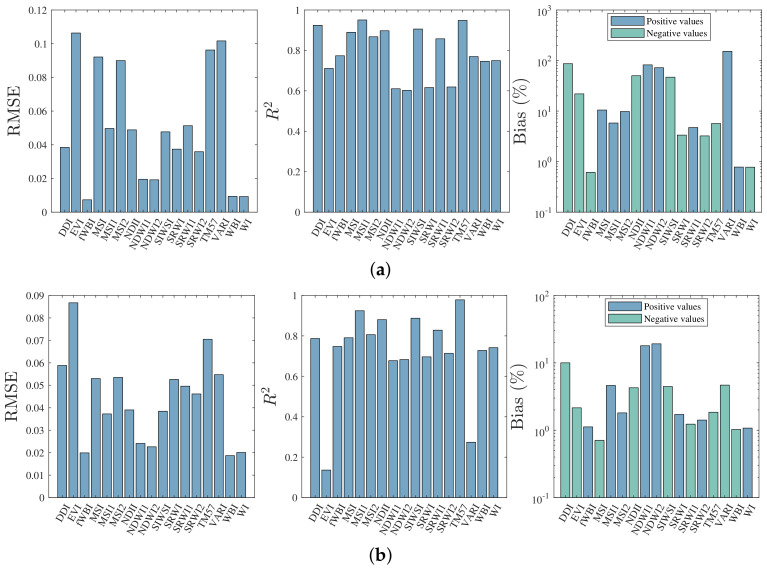
Evaluation of model performance for retrieval of vegetation indices. Where: (**a**) *Pinus radiata* and (**b**) *Eucalyptus globulus*.

The 17 remaining spectral indices were calculated following Equation (3). For both species, the $R^{2}$, the RMSE, and the percentage bias are depicted in Figure 3. The percentage bias was expressed with two different colors for positive and negative values—this distinction was made because the axis of the ordinates was logarithmic.

The spectral indices recovered from the LWI with the lowest RMSE were fWBI, WI, WBI, with values less than 0.0094 and 0.0201 for *Pinus* and *Eucalyptus*, respectively. On the other hand, the TM57 and MSI1 indices presented a coefficient of determination higher than 0.9244 in both species. Finally, considering the indices with lower RMSE and $R^{2}$ values; the WBI and WI indices had an absolute percentage bias lower than 1.07% for both species. It is worth noting that none of the wavelengths of the LWI formulation were contained in the five indices mentioned above.

In addition to these five vegetation indices, other spectral indices with significant evaluation metrics were obtained. In particular, for *Pinus radiata*, MSI, MSI2, NDII, SIWSI and SRWI1 with an RMSE was lower than 0.092, and the $R^{2}$ was higher than 0.85. On the other hand, for *Eucalyptus globulus*, the spectral indices MSI, MSI2, NDII, SIWSI and SRWI1 had an RMSE lower than 0.054, and $R^{2}$ higher than 0.791.

### 3.2. Retrieval of Non-Water Content Related VIs

After observing that the LWI was able to recover several vegetation indices related to water content, we used such an index to estimate other VIs not associated with moisture content (see Table 3). The results indicated that the LWI was suitable to estimate the VIs presented in Table 5 with an $R^{2}$ higher than 0.71, an RMSE lower than 0.0908 for *Pinus radiata*. For *Eucalyptus globulus*, the coefficient of determination is more elevated than 0.7475, and an RMSE lower than 0.0588.

## 4. Discussion

The assumption that the adjustment and validation sets are independent can be supported due to the difference in the mean spectrum of the two sets (see black line in Figure 2). Additionally, the spread of the individual reflectance spectra shows noticeable differences between the model and validation sets, as seen in Figure 2.

The relationship between the FMC and the eighteen VIs understudy is expressed as a coefficient of determination in Table 4. In general, the mean value of the $R^{2}$ coefficient, for the ten indices with the highest coefficient of determination, is 0.6723 and 0.7639 for *Pinus radiata* and *Eucalyptus globulus*, respectively. Such values suggest that given the correlation of spectral indices and water content, it would be possible to use only one to determine the others. The selection of the most suitable spectral index was made based on the coefficient of determination. The values obtained for RMSE, $R^{2}$, and bias suggest that it is possible to estimate several related spectral indices from a single one, in specific, Leaf Water Index (LWI). On the other hand, the results obtained when retrieving VIs that are not related to water content from the LWI point to the possibility of using such index to estimate a wide range of VIs; however, further work is needed in this area.

## 5. Conclusions

This study presents the findings on the potential of exploiting the relationship between FMC and vegetation indices to estimate the values of several moisture-related spectral indices form the knowledge of a single index. The work has focused on linear models, which are adjusted with data obtained in field sampling. The results suggest that it is possible to retrieve some vegetation indices from a Leaf Water Index (LWI). Specifically, the fWBI, WBI, WI can be recovered from the LWI up to an RMSE = 0.0201; $R^{2}$ = 0.77; and bias = 1.12% for both species. The main use of such a method is for the use of airborne sensors with discrete wavelengths instead of a continuous spectrum one to estimate vegetation indices.

## Figures and Tables

**Table 1:** Technical characteristics of the instruments.

Instrument	Specifications	
Spectrometer	Model	Terraspec 4 Hi-Res
	Manufacturer	ASD
	Range	350–2500 nm
	Spectral resolution	3 nm at 700 nm, 6 nm at 1400 nm
Scale	Model	PFB 120-3
	Manufacturer	KERN
	Max. weighing	120 g
	Reproducibility	0.001 g
Oven	Model	UN30
	Manufacturer	Memmert
	Range	20 ${}^{{}^{\circ}}$C to 30 ${}^{{}^{\circ}}$C
	Temperature accuracy	up to 99.9 ${}^{{}^{\circ}}$C: 0.1

**Table 2:** Vegetation indices used in the estimation of moisture content, including their acronym, name, formulation and source.

Acronym	Vegetation Index	Formulation	Source
DDI	Double Difference Index	$2R_{1530}-R_{1005}-R_{2055}$	[6,7]
EVI	Enhanced Vegetation Index	$2.5\left(R_{nir}-R_{red}\right)/\left(R_{nir}+6R_{red}-7.5R_{blue}+1\right)$	[11]
fWBI	Floating-position Water Band Index	$R_{900}/\min\left(R_{930}-R_{980}\right)$	[12]
LWI	Leaf Water Index	$R_{1300}/R_{1450}$	[13]
MSI	Moisture Stress Index	$R_{1600}/R_{820}$	[14]
MSI1	Moisture Stress Index 1	$R_{1650}/R_{1230}$	[15]
MSI2	Moisture Stress Index 2	$R_{1650}/R_{830}$	[15]
NDII	Normalized Difference Infrared Index	$\left(R_{850}-R_{1650}\right)/\left(R_{850}+R_{1650}\right)$	[16]
NDWI1	Normalized Difference Water Index 1	$\left(R_{860}-R_{1240}\right)/\left(R_{860}+R_{1240}\right)$	[17]
NDWI2	Normalized Difference Water Index 2	$\left(R_{870}-R_{1260}\right)/\left(R_{870}+R_{1260}\right)$	[18]
SIWSI	Shortwave Infrared Water Stress	$\left(R_{1640}-R_{858}\right)/\left(R_{1640}+R_{858}\right)$	[19]
SRWI	Simple Ratio Water Index	$R_{860}/R_{1240}$	[20]
SRWI1	Simple Ratio Water Index 1	$R_{1350}/R_{870}$	[18]
SRWI2	Simple Ratio Water Index 2	$R_{880}/R_{1265}$	[18]
TM57	Ratio of Thematic Mapper Band 5 to Band 7	$R_{1650}/R_{2220}$	[21]
VARI	Visible Atmospheric Resistant Index	$\left(R_{green}-R_{red}\right)/\left(R_{green}+R_{red}-R_{blue}\right)$	[22]
WBI	Water Band Index	$R_{970}/R_{900}$	[23]
WI	Water Index	$R_{900}/R_{970}$	[24]

**Table 3:** Vegetation indices non-related to the estimation of moisture content, including their acronym, name, formulation and source.

Acronym	Vegetation Index	Formulation	Source
NRI	Nitrogen reflectance index	$\left(R_{570}-R_{670}\right)/\left(R_{570}+R_{670}\right)$	[27]
ARI	Anthocyanin reflectance index	$\left(1/R_{550}\right)-\left(1/R_{700}\right)$	[28]
CI	Carotenoid Index	$R_{520}/R_{500}$	[29]
NDVI	Normalized Difference Vegetation Index	$\left(R_{nir}-R_{red}\right)/\left(R_{nir}+R_{red}\right)$	[30]

**Table 4:** Coefficient of determination for the FMC vs VI models using the fitting set.

Vegetation Index	*P. radiata*	*E. globulus*
DDI	0.5263	0.7379
EVI	0.2898	0.2061
fWBI	0.6286	0.6567
LWI	0.7742	0.8699
MSI	0.6668	0.7892
MSI1	0.6497	0.7637
MSI2	0.6566	0.7559
NDII	0.6599	0.7600
NDWI1	0.5702	0.6062
NDWI2	0.5643	0.6042
SIWSI	0.6681	0.7696
SRWI	0.5710	0.6068
SRWI1	0.7321	0.7818
SRWI2	0.5733	0.6146
TM57	0.6450	0.7497
VARI	0.5101	0.1525
WBI	0.6359	0.6606
WI	0.6344	0.6616

**Table 5:** Evaluation of Leaf Water Index (LWI) to retrieve non-water content related vegetation indices (VIs).

	*Pinus radiata*			*Eucalyptus globulus*		
	**RMSE**	$R^{2}$	**Bias (%)**	**RMSE**	$R^{2}$	**Bias (%)**
NRI	0.0338	0.9243	−75.9749	0.0588	0.7870	−9.9942
ARI	0.0073	0.7735	−0.6143	0.0201	0.7475	1.0940
CI	0.0898	0.8898	10.2368	0.0530	0.7908	−0.6564
NDVI	0.0464	0.9507	5.4369	0.0364	0.9244	4.5324

**Figure A1:**
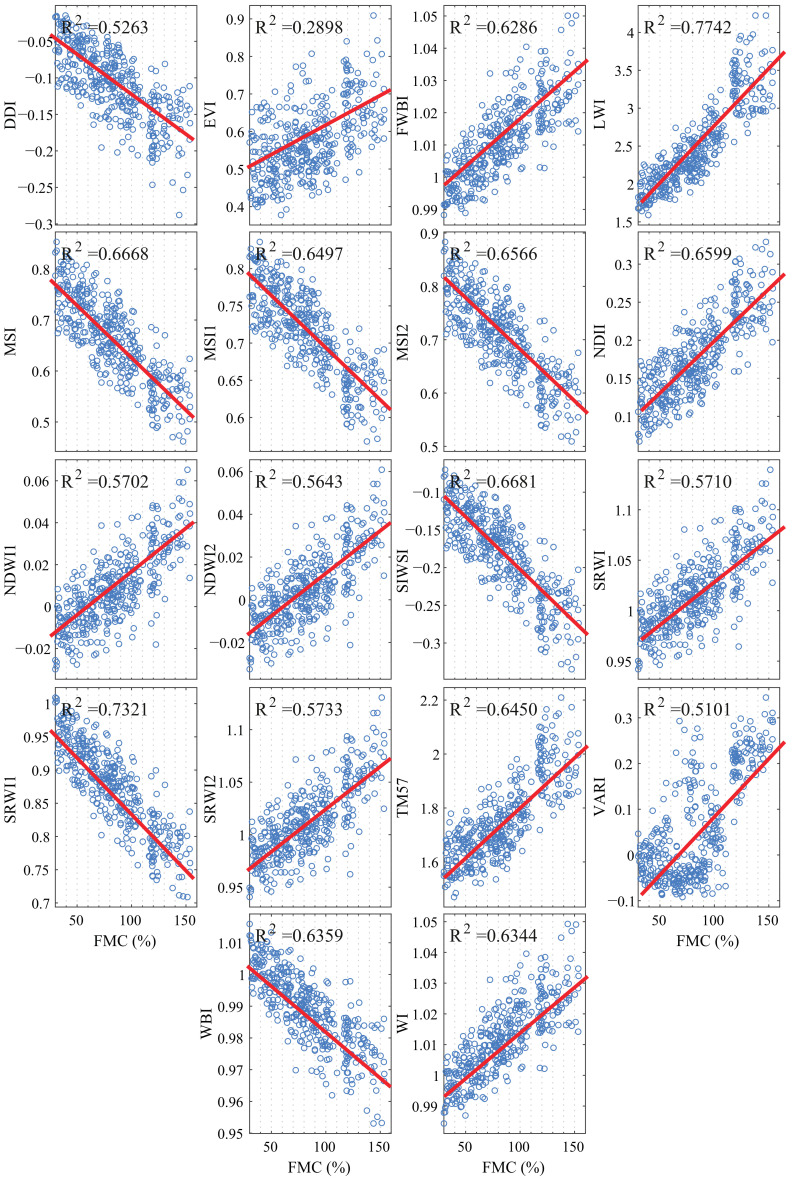
VIs variation as function of Fuel Moisture Content for *Pinus radiata*.

**Figure A2:**
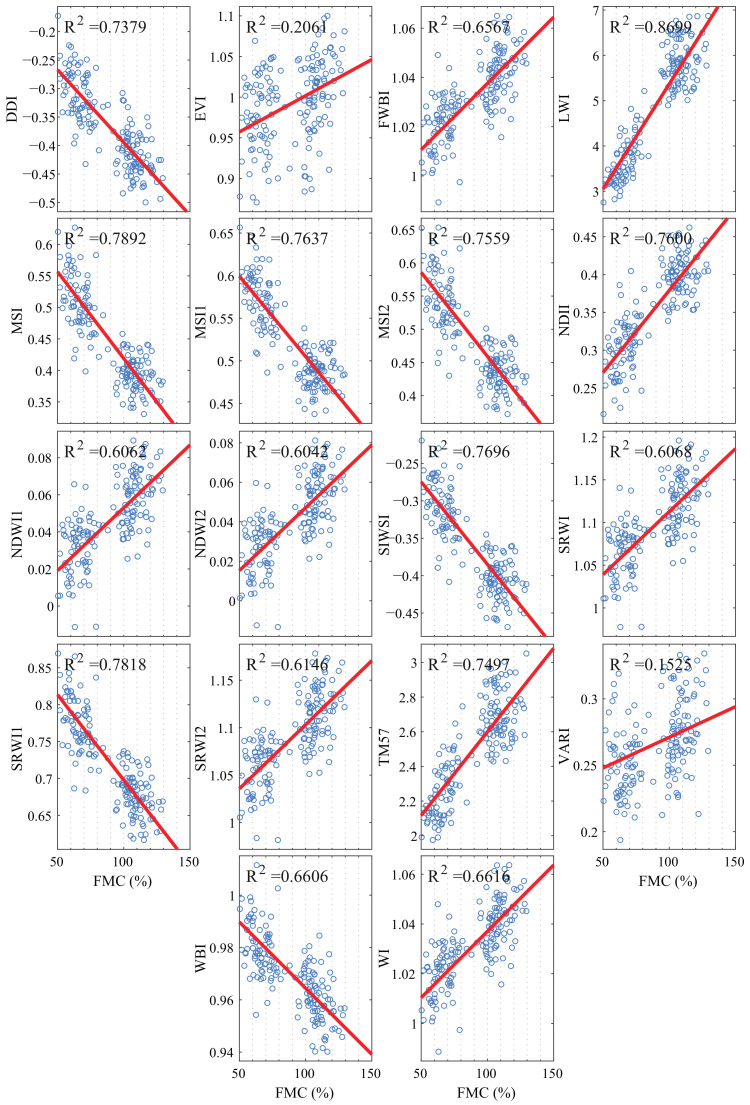
VIs variation as function of Fuel Moisture Content for *Eucalyptus globulus*.

## Data Availability

The data presented in this report are available on request from the corresponding author.

## Abbreviations

The following abbreviations are used in this manuscript:

EM	Electromagnetic
FMC	Fuel Moisture Content
$R^{2}$	Coefficient of determination
VI	Vegetation index
